# Procalcitonin for diagnosis of bacterial pneumonia in critically ill patients during 2009 H1N1 influenza pandemic: a prospective cohort study, systematic review and individual patient data meta-analysis

**DOI:** 10.1186/cc13760

**Published:** 2014-03-10

**Authors:** Roman Pfister, Matthias Kochanek, Timo Leygeber, Christian Brun-Buisson, Elise Cuquemelle, Mariana Benevides Paiva Machado, Enrique Piacentini, Naomi E Hammond, Paul R Ingram, Guido Michels

**Affiliations:** 1 Department of Internal Medicine III, University of Cologne, Kerpener Str. 62, 50937 Cologne, Germany; 2 Department of Internal Medicine I, University of Cologne, Cologne, Germany; 3 Medical Intensive Care Unit, Hôpitaux Universitaires Henri Mondor, Assistance Publique-Hôpitaux de Paris, Université Paris-Est Créteil, Créteil, France; 4 Hospital das Clínicas, Universidade Federal de Minas Gerais, Belo Horizonte, Brazil; 5 Department of Critical Care Medicine, Hospital Universitari Mutua Terrassa, Terrassa, Spain; 6 Department of Intensive Care Medicine, Royal North Shore Hospital, Sydney, Australia; 7 The George Institute for Global Health, Sydney, Australia; 8 Department of Microbiology and Infectious Diseases, Royal Perth Hospital, Perth, Australia; 9 School of Pathology and Laboratory Medicine, University of Western Australia, Perth, Australia

## Abstract

**Introduction:**

Procalcitonin (PCT) is helpful for diagnosing bacterial infections. The diagnostic utility of PCT has not been examined thoroughly in critically ill patients with suspected H1N1 influenza.

**Methods:**

Clinical characteristics and PCT were prospectively assessed in 46 patients with pneumonia admitted to medical ICUs during the 2009 and 2010 influenza seasons. An individual patient data meta-analysis was performed by combining our data with data from five other studies on the diagnostic utility of PCT in ICU patients with suspected 2009 pandemic influenza A(H1N1) virus infection identified by performing a systematic literature search.

**Results:**

PCT levels, measured within 24 hours of ICU admission, were significantly elevated in patients with bacterial pneumonia (isolated or coinfection with H1N1; *n* = 77) (median = 6.2 μg/L, interquartile range (IQR) = 0.9 to 20) than in patients with isolated H1N1 influenza pneumonia (*n* = 84; median = 0.56 μg/L, IQR = 0.18 to 3.33). The area under the curve of the receiver operating characteristic curve of PCT was 0.72 (95% confidence interval (CI) = 0.64 to 0.80; *P* < 0.0001) for diagnosis of bacterial pneumonia, but increased to 0.76 (95% CI = 0.68 to 0.85; *P* < 0.0001) when patients with hospital-acquired pneumonia and immune-compromising disorders were excluded. PCT at a cut-off of 0.5 μg/L had a sensitivity (95% CI) and a negative predictive value of 80.5% (69.9 to 88.7) and 73.2% (59.7 to 84.2) for diagnosis of bacterial pneumonia, respectively, which increased to 85.5% (73.3 to 93.5) and 82.2% (68.0 to 92.0) in patients without hospital acquired pneumonia or immune-compromising disorder.

**Conclusions:**

In critically ill patients with pneumonia during the influenza season, PCT is a reasonably accurate marker for detection of bacterial pneumonia, particularly in patients with community-acquired disease and without immune-compromising disorders, but it might not be sufficient as a stand-alone marker for withholding antibiotic treatment.

## Introduction

In 2009, the pandemic influenza A(H1N1) virus infection caused more than 18,000 deaths and was associated with more than 200,000 estimated respiratory deaths worldwide [1]. Although most H1N1 patients in 2009 and 2010 presented with mild febrile illnesses and cough, sore throat and occasional gastrointestinal symptoms, 7% to 25% of patients developed severe respiratory distress requiring admission to ICUs [2-5]. Bacterial coinfection in ICU patients with 2009 H1N1 influenza infection is reported to be 18% to 34% and is associated with significantly increased mortality [6,7]. Importantly, the initial clinical features of isolated H1N1 infection cannot be distinguished from additional bacterial coinfection [6]. Antibiotic treatment is not recommended in patients with H1N1 infections, but early antibiotic treatment in patients with community-acquired pneumonia is associated with decreased mortality [8]. A rapid diagnosis at admission of bacterial coinfection or isolated bacterial infection is crucial to early initiation or withdrawal of antibiotic treatment. However, obtaining the results cultures for bacterial pathogens may take several days. As a consequence, researchers in several studies have analyzed the predictive value of biomarkers to distinguish bacterial from isolated viral pneumonia [9,10].

Procalcitonin (PCT) may be a clinically useful marker for the diagnosis of bacterial infections [11,12] and may be a useful adjunct for reducing antibiotic usage [13-15]. However, data on the diagnostic value of PCT, including comparisons with other biomarkers such as C-reactive protein (CRP), in critically ill patients in the setting of 2009 H1N1 influenza infection are rare, and the reported results are inconsistent [16-20]. Earlier results of studies on PCT in critically ill patients might not be transferable to pandemic H1N1 influenza, because the latter is associated with a remarkable release of proinflammatory cytokines such as interleukin 6 and a defective T-cell response, both of which might affect PCT release and hence discriminatory performance of PCT in diagnosing bacterial infection [21,22]. Conflicting results of studies of PCT in critically ill patients with suspected 2009 H1N1 influenza are most likely due to the small sample sizes and substantial differences in populations studied. More powerful analyses are needed to address the role of potential confounders of the diagnostic accuracy of PCT, such as antibiotic pretreatment, underlying impairment of the immune system and hospital-acquired infections.

Our aims in the present study were to test the utility of PCT in the diagnosis of bacterial pneumonia in patients admitted to the ICU during the 2009 H1N1 influenza pandemic, including its performance in important clinical subgroups, and to compare its performance with CRP. We performed a prospective cohort study in patients admitted to a 28-bed ICU of a tertiary care hospital with pneumonia during the 2009 and 2010 influenza seasons and then combined our data with individual patient data derived from published studies identified by conducting a systematic literature search and performed a meta-analysis.

## Material and methods

### Prospective cohort study

The study population was patients admitted to a medical ICU at a tertiary care hospital in Cologne, Germany, during the pandemic influenza A(H1N1) season between November 2009 and January 2010 as well as those admitted between December 2010 and May 2011. All adult patients (older than 18 years of age) with community-acquired pneumonia and visual evidence of a pulmonary infiltrate on a chest X-ray or computed tomography scan were included. In addition, we included patients with hospital-acquired pneumonia admitted to the ICU during a local outbreak of H1N1 influenza within our hospital during the study time periods. *Hospital-acquired pneumonia* was defined as disease that manifested later than 48 hours after hospital admission. H1N1 status was assessed by PCR in every patient using nasopharyngeal swabs or bronchoalveolar lavage fluid upon admission to the ICU. Standard diagnostic tests for bacterial pathogens were performed as part of the routine clinical assessment universally applied to all consecutive patients. *Bacterial pneumonia* was defined by detection of a bacterial pathogen compatible with pneumonia infection using blood cultures, urinary antigen tests (for *Pneumococcus* and *Legionella*), PCR (for *Chlamydia* and *Mycoplasma*) and/or cultures of respiratory tract secretions (tracheobronchial aspirate or bronchoalveolar lavage). PCT and CRP were measured using the ELECSYS B•R•A•H•M•S PCT electrochemiluminescence assay (Fisher Scientific, Schwerte, Germany) and the Tina-Quant C-Reactive Protein Gen 3 assay (Roche Diagnostics, Mannheim, Germany) according to the treating physician’s discretion. Impairment of the immune system was defined by the presence of active leukemia or lymphoma or if myelotoxic chemotherapy was applied. The study was approved by the ethics committee of the medical faculty of the University of Cologne, and informed consent was waived because all data assessed in the study were part of routine clinical practice and retrieved from patient records without any study intervention.

### Systematic review

In accordance with PRISMA recommendations (Preferred Reporting Items for Systematic Reviews and Meta-Analyses) [23], we performed an electronic literature search using PubMed to identify studies reporting the performance of PCT in differentiating 2009 H1N1 influenza from bacterial pneumonia in adult ICU patients. Restrictions regarding language and publication year were not applied, and publications up until June 2013 were included. The following search terms were used: “procalcitonin” in combination with “H1N1,” “influenza,” “swine flu” or “avian flu.” Two independent reviewers (TL and RP) screened the electronic search results for eligible articles by reading the titles and abstracts. Inclusion criteria for in-depth examinations were the evaluation of PCT alone or in comparison with other laboratory markers, such as CRP, to diagnose H1N1 influenza or bacterial pneumonia in adult patients with respiratory tract infections admitted to ICUs. References to other sources within the selected articles were researched for other eligible articles.

For all eligible articles, we contacted the authors by e-mail and invited them to share their data in this collaborative project. Data sets needed to include at least the following variables: anonymous patient identifiers and patient characteristics obtained from clinical history (gender, age, antibiotics given before ICU admission, community- or hospital-acquired pneumonia, underlying impairment of the immune system and results of testing for H1N1 and bacteria). Because individual studies used different risk scores to assess the severity of critical illness and mortality, we defined *predicted high risk* using cutoffs of respective risk scores which might be associated with an in-hospital mortality rate greater than 15%: Sequential Organ Failure Assessment score higher than 7, Simplified Acute Physiology Score II (SAPS II) higher than 35, SAPS III score above 47 and Acute Physiology and Chronic Health Evaluation II score higher than 15.

All individual studies were approved by the respective local ethics committees where appropriate (Ethics Review Board of the Société de Réanimation de langue Française [16], the ethical committee of Hospital Universitario Mútua de Terrassa [20], the Prince Charles Hospital Human Research Ethics Committee [17] and the ethics committee of Universidade Federal de Minas Gerais [19]). Approval was not required in the study by Ingram *et al*. [18].

### Statistical analysis

We merged the data of our cohort and the previously published cohorts into a summary database if variables were compatible. Incompatible data were first recoded and then added to the summary database. We included into the analysis only patients with PCT measured within the first 24 hours after admission to the ICU and with either confirmed H1N1 influenza or microbiologically confirmed bacterial pneumonia, or both. If more than one measure of PCT or CRP was available within 24 hours after admission, we used the first measure performed after admission for analysis. Continuous variables are expressed as median and interquartile range (IQR) or as mean ± standard deviation (SD) as appropriate. Categorical variables are presented as absolute and relative frequencies. Pairwise comparisons for categorical variables were performed by using a χ2 test. For continuous variables, we used a nonparametric Mann–Whitney *U* test. The diagnostic discrimination of inflammatory markers for diagnosis of microbiologically confirmed bacterial pneumonia (combining patients with isolated bacterial pneumonia with patients with mixed bacterial and H1N1 pneumonia) from isolated H1N1 pneumonia is expressed by receiver operating characteristic curve (ROC) data. Sensitivity, specificity, positive predictive value and negative predictive value were calculated. Because distinct population-specific issues might influence the discriminatory performance of PCT, we performed sensitivity analyses in subgroups without impairment of the immune system, with community-acquired pneumonia, with typical bacterial pathogens (excluding *Mycoplasma*, *Chlamydia* and *Legionella* infections), without extracorporeal membrane oxygenation (ECMO) and renal replacement therapy and without antibiotics given before ICU admission. *P*-values less than 0.05 were considered to be statistically significant. All reported *P*-values are two-sided, except for comparison of areas under the curve (AUCs). Statistical analyses were performed with Stata/SE 12.1 for Windows software (StataCorp, Austin, TX, USA) and SPSS Statistics version 21 software (IBM SPSS, Chicago, IL, USA).

## Results

### Prospective cohort study

Table 1 shows the baseline characteristics of our cohort. The mean age (±SD) of the patients was 56 (±17) years, and 50% of the patients were male. Sixty-one percent of the patients had bacterial pneumonia, 33% were being treated with antibiotics before ICU admission and 57% were positive for H1N1. Overall, 67% of the patients had hospital-acquired pneumonia and 35% had immune-compromising disorders. All patients had at least one measure of PCT within the first 24 hours after admission to the ICU.

**Table 1:** **Characteristics of our prospective cohort study**^**a**^

**Characteristics**	**Patient data**
	**(*n*** **= 46)**
Gender (male)	23 (50%)
Age (years), mean (±SD)	56 ± 17
H1N1 status	26 (57%)
Death in ICU	15 (33%)
TISS-28, median (IQR)^b^	21 (19 to 25)
SAPS II, median (IQR)^b^	51 (41 to 60)
APACHE II, median (IQR)^b^	22 (17 to 26)
SOFA, median (IQR)^b^	9 (7 to 11)
Asthma	2 (4.3%)
COPD	11 (24%)
Smokers	9 (20%)
Alcoholic disease	5 (11%)
Diabetes mellitus	12 (26%)
Immune-compromising disorder	16 (35%)
Hospital-acquired pneumonia	31 (67%)
Bacterial pathogen	28 (61%)
*Pneumococcus*	6
*Chlamydia*/*Mycoplasma*	3
*Legionella*	2
Gram-negative bacilli (*Pseudomonas aeruginosa*, *Klebsiella*, *Morganella morganii*)	9
*Staphylococcus aureus*	4
Other gram-positive cocci (*Enterococci*, coagulase-negative *Staphylococci*)	4
Antibiotics given during ICU	31 (67%)
Antibiotics given before ICU	15 (33%)
Mechanical ventilation	34 (74%)
Length of ventilation (days), median (IQR)	7 (1 to 11)
ECMO	5 (11%)
Renal replacement therapy	9 (19.6%)
Vasopressor therapy	30 (65%)
Length of stay (days), median (IQR)	9 (5 to 16)
Procalcitonin (μg/L) at admission (*n* = 36)	0.85 (0.3 to 4.1)
Procalcitonin (μg/L) within day 1 (*n* = 30)	0.9 (0.2 to 6)
C-reactive protein (mg/L) at admission (*n* = 40)	159 (61 to 253)
C-reactive protein (mg/L) within day 1 (*n* = 30)	101 (62 to 208)

^a^APACHE II, Acute Physiology and Chronic Health Evaluation II; COPD, Chronic obstructive pulmonary disease; ECMO, Extracorporeal membrane oxygenation; IQR, Interquartile range; SAPS II, Simplified Acute Physiology Score II; SD, Standard deviation; SOFA, Sequential Organ Failure Assessment; TISS-28, 28-item Therapeutic Intervention Scoring System. ^b^Assessed at ICU admission.

### Systematic review

**Figure 1:**
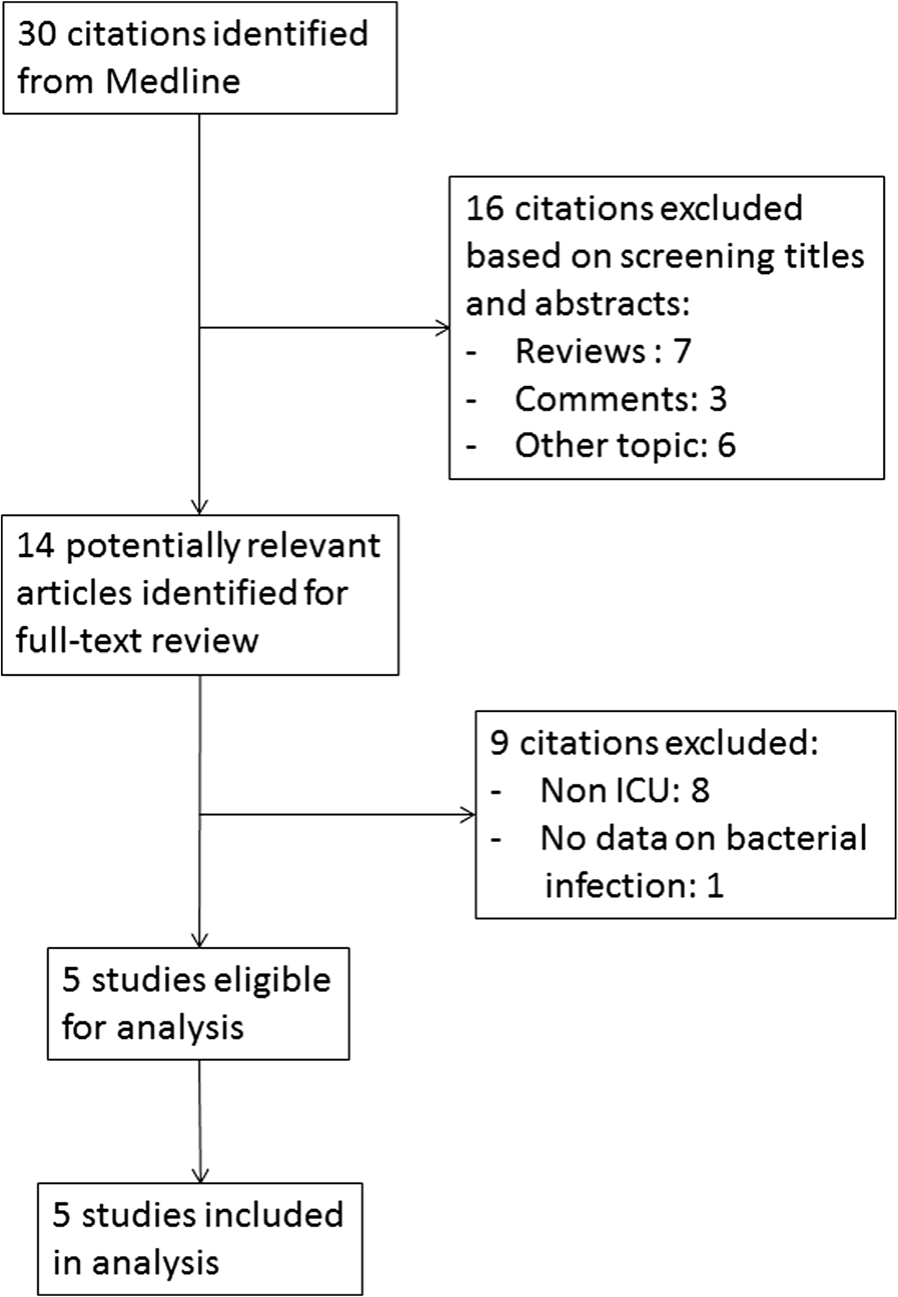
Flowchart used for systematic literature search.

A flowchart depicting the literature search process and inclusion of studies for our meta-analysis is shown in Figure 1. We identified 30 potentially relevant titles on MEDLINE that reported on utilizing PCT in adult patients with suspected influenza pneumonia. From among these 30 articles, 16 articles were excluded after the abstract was read because they were reviews without original data (*n* = 7), were comments without original data (*n* = 3) or were focused on other topics (for example, pediatric patients and outcomes without information on bacterial infection or vaccination; *n* = 6). Upon reading the full text of the remaining 14 studies, we found that 8 studies were on non-ICU patients and one did not provide data on bacterial infection; therefore, they were excluded, leaving us with 5 studies for analysis. Checks of for cross-references of selected articles identified no other applicable studies. The authors of all five included studies responded after we contacted them by e-mail, and they sent us their primary data. The characteristics of these five studies are shown in Table 2. The authors in all five of the included studies examined patients with community-acquired pneumonia, whereas we examined mixed community- and hospital-acquired pneumonia patients in our study.

**Table 2:** **Characteristics of studies included in the meta-analysis**^**a**^

**Study**	**Year**	**PCT available *n*/N (assay)**	**Study design**	**Inclusion criteria**	**H1N1-positive (%)**^**b**^	**Bacterial infection (%)**^**b**^	**Hospital-acquired pneumonia (%)**^**b**^	**Antibiotics before admission (%)**^**b**^	**Immune disorder (%)**^**b**^
Piacentini *et al*. [20]	2010	22/22 (not reported)	Prospective	Adults, ICU, community-acquired pneumonia	45	50 (cultures, antigen)	0	100	5
Hammond *et al*. [17]	2011	17/17 (VIDAS B•R•A•H•M•S PCT)	Retrospective	Adults, ICU, respiratory tract infection	82	41 (cultures)	0	n.a.	0
Cuquemelle *et al*. [16]	2011	52/103 (B•R•A•H•M•S KRYPTOR PCT)	Prospective	Adults, ICU, community-acquired pneumonia, H1N1-positive	100	37 (cultures, antigen)	0	0	6
Ingram *et al*. [18]	2010	16/25 (VIDAS B•R•A•H•M•S PCT)	Retrospective	Adults, ICU, community-acquired pneumonia, positive for H1N1 or bacterial infection	63	38 (cultures, seroconversion)	0	94	6
Paiva *et al*. [19]	2012	29/29 (VIDAS B•R•A•H•M•S PCT)	Prospective	Adults, ICU, pneumonia, suspected H1N1	41	21 (cultures)	0	41	14

^a^PCT, Procalcitonin. ^b^Referring to patients with available procalcitonin.

When we combined our cohort with the data derived from the other five studies, we had a total of 242 patients. We excluded 60 patients with missing PCT measured within the first day of ICU admission and 21 patients with neither confirmation of H1N1 influenza infection nor confirmation of a bacterial pathogen causative of infection. Thus we had 161 patients remaining for inclusion in the analyses. Patients who were excluded (*n* = 81) did not differ significantly regarding age, immune-compromising disorder, H1N1 status, bacterial infection rate or CRP level (all *P* > 0.05); however, compared to patients who were included, the excluded group had a higher ratio of women, with borderline statistical significance (60% vs. 46%; *P* = 0.03), and a lower ratio of antibiotics given before ICU admission (19% vs. 38%; *P* = 0.004).

Table 3 shows the characteristics of the study population divided by bacterial infection status. Eighty-four patients had isolated H1N1 infections and seventy-seven had bacterial infections (sixty-nine with typical bacterial pathogens and eight with atypical bacterial pathogens), with thirty-seven patients having isolated bacterial infections and forty having mixed H1N1 and bacterial infections. One hundred forty-three patients also had CRP measured within the first 24 hours after admission. The baseline characteristics did not differ significantly between patients with vs. without bacterial infections, except for H1N1 status, high-risk of mortality predicted on the basis of ICU risk scores and the inflammatory markers PCT and CRP.

**Table 3:** **Baseline characteristics of the pooled study population by status of bacterial infection**^**a**^

**Characteristics**	**Isolated H1N1 infection**	**Bacterial infection**
*N*	84	77
Age (years), mean (±SD)	43.9 (18.6)	47.6 (18.8)
Gender, male	48 (57%)	39 (51%)
Hospital-acquired	12 (14%)	18 (23%)
Antibiotics given before ICU^b^	30 (39%)	25 (36%)
Immune-compromising disorder	10 (12%)	11 (14%)
H1N1 status	84 (100%)	40 (52%)^d^
In-hospital mortality	17 (20.2%)	15 (19.5%)
Predicted high risk^b^	34 (41.0%)	49 (67.1%)^d^
Mechanical ventilation	54 (64.3%)	57 (74.0%)
Vasopressor therapy^b^	32 (48.5%)	34 (53.1%)
ECMO	10 (11.9%)	8 (10.4%)
Renal replacement therapy^b^	10 (13.3%)	16 (22.9%)
Procalcitonin (μg/L), median (IQR)	0.56 (0.18 to 3.3)	6.2 (0.9 to 30)^d^
C-reactive protein (mg/L) (median (IQR)^c^	108 (50 to 213)	214 (81 to 335)^d^

^a^ECMO, Extracorporeal membrane oxygenation; IQR, Interquartile range; SD, Standard deviation. ^b^Data do not add up to 161 because of missing data for some individuals. Percentage data refer to individuals with available data. ^c^Available for 143 of 161 patients. ^d^*P* < 0.05 for comparisons between groups.

**Figure 2:**
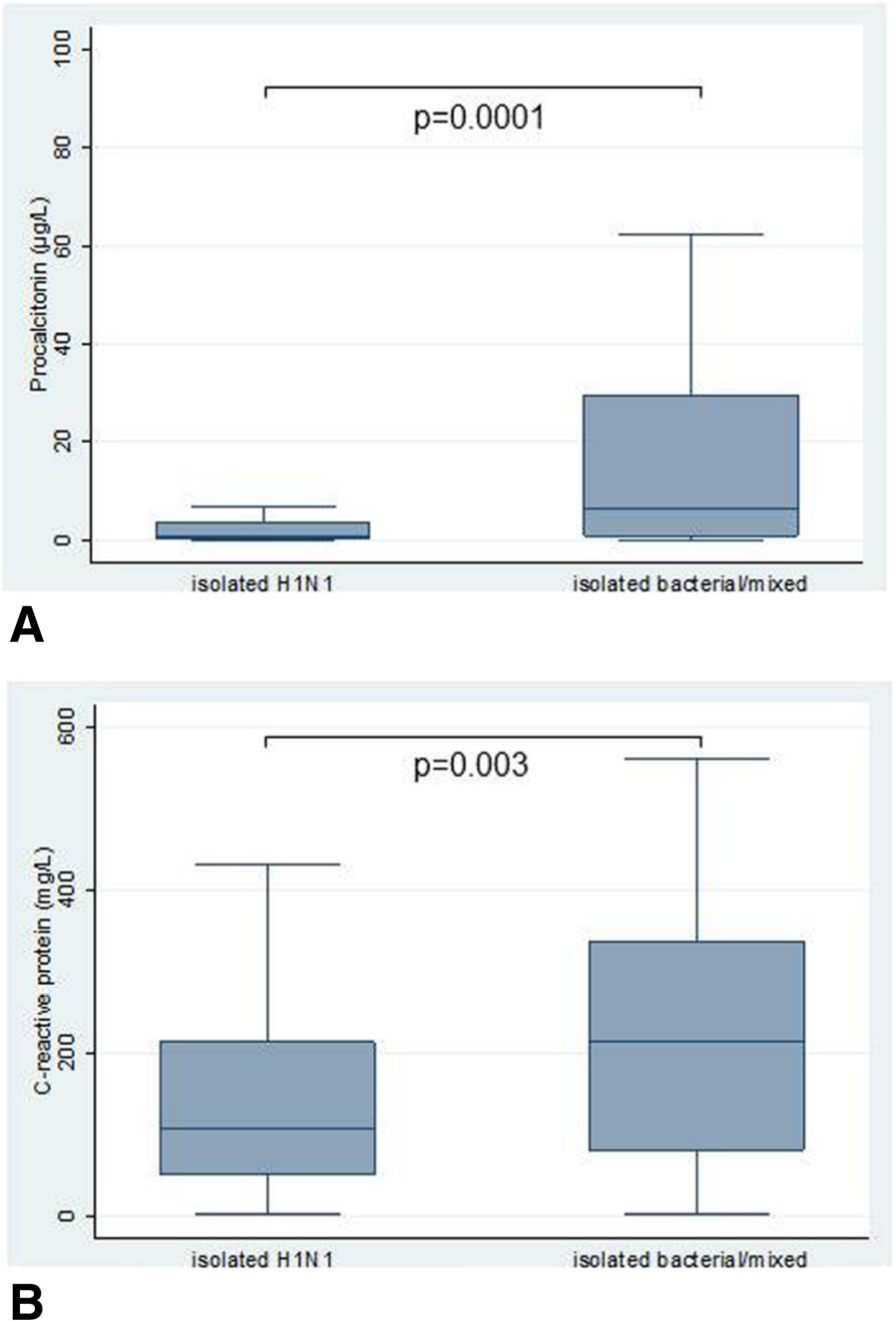
**Procalcitonin and C-reactive protein levels by status of bacterial pneumonia (isolated bacterial or mixed bacterial and H1N1 infection versus isolated H1N1). (A)** Procalcitonin. **(B)** C-reactive protein box representing interquartile range, line subdividing box representing median, whisker span all values within 1.5 interquartile ranges of the nearer quartile.

Figure 2A shows PCT and CRP levels by status of bacterial infection. Patients with bacterial pneumonia had significantly elevated PCT levels (6.2 μg/L; IQR = 0.9 to 20) compared to those with isolated H1N1 pneumonia (0.56 μg/L; IQR = 0.18 to 3.3, *P* = 0.0001). There was no significant difference in PCT levels between patients with isolated bacterial pneumonia and mixed bacterial and H1N1 pneumonia (*P* = 0.14), as shown in Additional file 1. Both isolated bacterial pneumonia patients (3.63 μg/L; IQR = 0.63 to 18.2, *P* = 0.002) and mixed bacterial and H1N1 pneumonia patients (11.36 μg/L; IQR = 1.07 to 41.14, *P* = 0.0001) had significantly elevated PCT levels compared to those with isolated H1N1 pneumonia (0.56 μg/L; IQR = 0.18 to 3.33) (see Additional file 1). Patients with bacterial pneumonia had significantly elevated CRP levels (214 mg/L; IQR = 81 to 335) compared with patients with isolated H1N1 pneumonia (108 mg/L; IQR = 50 to 213, p = 0.003) (Figure 2B). Neither PCT levels (2.21 μg/L; IQR = 0.55 to 12.6 vs. 1.2 μg/L; IQR = 0.3 to 11.9, *P* = 0.34) nor CRP levels (175 mg/L; IQR = 77 to 311 vs. 142 mg/L, IQR = 58 to 278, *P* = 0.14) were significantly different between patients who died during their hospital stay and those who survived.

**Figure 3:**
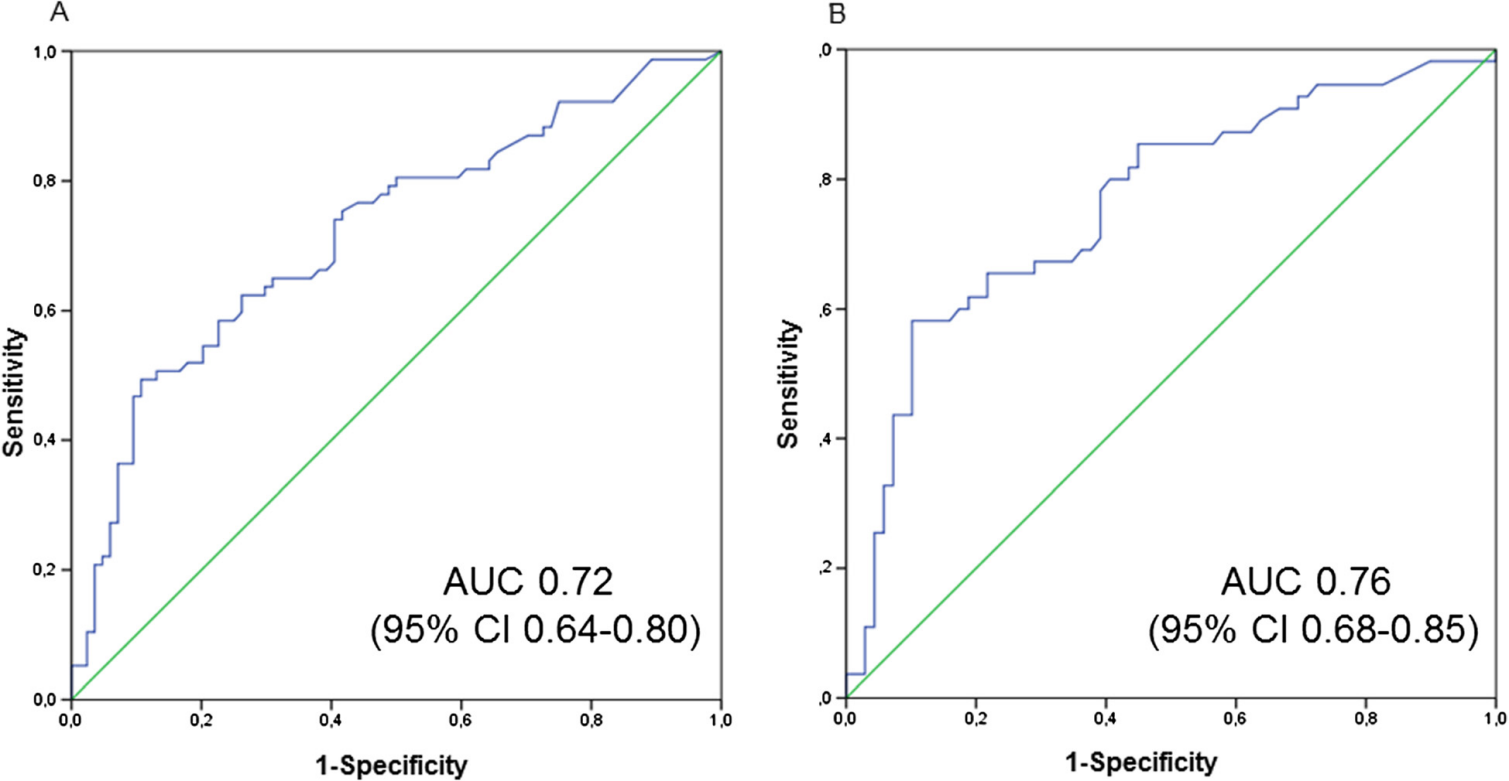
**Area under the receiver operating characteristic curves for detection of bacterial pneumonia by procalcitonin. (A)** Area under the receiver operating characteristic curve (AUC) with 95% confidence interval (CI) representing data from the total study population. **(B)** AUC with 95% CI representing data from patients without hospital-acquired pneumonia or immune-compromising disease.

Figure 3 shows the ROC curves and Table 4 shows the discriminatory performance of PCT for detection of bacterial pneumonia. The AUC of the ROC curve of PCT in the total population was 0.72 (95% CI = 0.64 to 0.80; *P* < 0.0001). The results were similar when only PCT levels measured immediately upon admission to the ICU were considered (data not shown). When we restricted the analysis to bacterial infections with typical pathogens, excluding *Mycoplasma*, *Chlamydia* and *Legionella*, the AUC was 0.73 (95% CI = 0.64 to 0.81; *P* < 0.0001). Exclusion of patients who received antibiotic treatment before ICU admission or patients with ECMO or renal replacement therapy did not change the discriminatory performance of PCT substantially. When we excluded patients with hospital-acquired pneumonia and patients with immune-compromising disorders, the AUC increased to 0.76 (95% CI = 0.68 to 0.85; *P* < 0.0001), which was not significantly different from the overall population (*P*-value for comparison = 0.24). The discriminatory performance of CRP assessed according to the AUC (0.64; 95% CI = 0.55 to 0.73) was lower than that of PCT, which was of borderline statistical significance (*P* = 0.10).

**Table 4:** **Discriminatory accuracy of procalcitonin and C-reactive protein for detection of bacterial pneumonia**^**a**^

**Characteristics**	**AUC**	**95% CI**	***P*-values**	***P*-value vs. total population**
Total (*N* = 161)	0.72	0.64 to 0.80	<0.0001	–
Without antibiotics before admission (*n* = 91)	0.73	0.62 to 0.83	<0.0001	0.47
Without ECMO or renal replacement therapy (*n* = 110)	0.74	0.64 to 0.83	<0.0001	0.40
Without hospital-acquired pneumonia (*n* = 131)	0.76	0.68 to 0.84	<0.0001	0.25
Without immune-compromising disorder (*n* = 140)	0.74	0.65 to 0.82	<0.0001	0.40
Without hospital-acquired pneumonia and	0.76	0.68 to 0.85	<0.0001	0.24
Without immune-compromising disorder (*n* = 124)				
CRP (total population, *n* = 143)	0.64	0.55 to 0.73	0.003	0.10

^a^AUC, Area under the curve; CI, 95% confidence interval; CRP, C-reactive protein; ECMO, extracorporeal membrane oxygenation.

A cutoff PCT level of 0.5 μg/L showed a sensitivity and negative predictive value of 80.5% (95% CI = 69.9 to 88.7) and 73.2% (95% CI = 59.7 to 84.2), respectively, for detection of a bacterial pneumonia (Table 5). The accuracy improved when we excluded patients with hospital-acquired pneumonia or immune-compromising disorder, with a respective sensitivity and negative predictive value of 85.5% (95% CI = 73.3 to 93.5) and 82.2% (95% CI = 68.0 to 92.0).

**Table 5:** **Discriminatory performance of procalcitonin for detection of bacterial pneumonia**^**a**^

	**Cutoff**	**Sensitivity, % (95% CI)**	**Specificity, % (95% CI)**	**Positive PV, % (95% CI)**	**Negative PV, % (95% CI)**	**Positive likelihood ratio (95% CI)**	**Negative likelihood ratio, % (95% CI)**
Total	>0.25 μg/L	87.0 (77.4 to 93.6)	28.6 (19.2 to 39.5)	52.8 (43.7 to 61.7)	70.6 (52.5 to 84.9)	1.22 (1.04 to 1.43)	0.45 (0.23 to 0.89)
	>0.5 μg/L	80.5 (69.9 to 88.7)	48.8 (37.7 to 60.0)	59.1 (49.0 to 68.6)	73.2 (59.7 to 84.2)	1.57 (1.24 to 1.99)	0.40 (0.24 to 0.66)
	>1 μg/L	71.4 (60.0 to 81 to 2)	59.5 (48.3 to 70.1)	61.8 (50.9 to 71.9)	69.4 (57.5 to 79.8)	1.76 (1.31 to 2.37)	0.48 (0.32 to 0.71)
Without hospital-acquired pneumonia or immune-compromising disorder	>0.25 μg/L	90.9 (80.1 to 97.0)	31.9 (21.2 to 44.2)	51.6 (41.2 to 61.8)	81.5 (61.9 to 93.7)	1.33 (1.11 to 1.60)	0.29 (0.12 to 0.70)
	>0.5 μg/L	85.5 (73.3 to 93.5)	53.6 (41.2 to 65.7)	59.5 (47.9 to 70.4)	82.2 (68.0 to 92.0)	1.84 (1.40 to 2.43)	0.27 (0.14 to 0.53)
	>1 μg/L	74.6 (61.0 to 86.3)	60.9 (48.4 to 72.4)	60.3 (47.7 to 72.0)	75.0 (61.6 to 85.6)	1.91 (1.37 to 2.66)	0.42 (0.26 to 0.68)

^a^CI, Confidence interval; PV, Predictive value.

## Discussion

By utilizing an individual patient data meta-analytic approach in this study, we show that, among patients admitted to the ICU with pneumonia during the H1N1 pandemics in 2009 and 2010, PCT levels were significantly increased in patients with bacterial pneumonia compared to those with isolated H1N1 pneumonia. The overall discriminatory performance of PCT for identification of bacterial pneumonia was moderate, with an AUC of 0.72, and seemed to be negatively affected by the presence of hospital-acquired infections and immune-compromising disorders. The AUC increased to 0.76 when these conditions were excluded from the analysis, and PCT at a cutoff of 0.5 μg/L had a sensitivity and negative predictive value of 85.5% (95% CI = 73.3 to 93.5) and 82.2% (95% CI = 68.0 to 92.0), respectively.

Patients with 2009 H1N1 infection and potential bacterial coinfection admitted to an ICU are at high risk for mortality [3-6,24]. Early differentiation of isolated viral infection from isolated or associated bacterial infection might help in the decision to initiate appropriate antimicrobial treatment or avoid unnecessary treatment. To date, studies evaluating PCT in ICU patients with suspected or confirmed 2009 H1N1 influenza have included between 16 and 52 patients, and, accordingly, they have major inherent limitations [16-20]. Owing to small sample sizes, differences in inclusion criteria, patient selection and outcome definitions, the discriminatory capacity of PCT appears to be unclear [17,19], with researchers in some studies reporting good discriminatory performance of PCT [16,18,20]. Authors of a recent meta-analysis examined the value of PCT for the detection of bacterial coinfection in patients with influenza [25]. Of note, only two of the six studies included in that meta-analysis exclusively comprised ICU patients. Both of these studies were included in our analyses [16,18]. Additionally, the authors of that meta-analysis did not use individual patient data, but instead reported only meta-analysis of summary statistics such as AUC, sensitivity and specificity.

Our collaborative approach yielded a sample of 161 patients, which is three times larger than any earlier study on this ICU collective and allowed us to estimate discriminatory performance more robustly than other studies, not only in the overall population but also in important subgroups. We demonstrate improved accuracy when patients with compromised immune systems and hospital-acquired disease are excluded, albeit that this difference did not reach statistical significance, most likely because of the limited sample size. The first finding might be explained by a trend toward overall lower PCT levels in immune-compromised patients [26]. Regarding the latter point, PCT is known to have weaker diagnostic utility in patients with nosocomial pneumonia because hospitalized patients generally have higher exposure to bacterial infections independently of potential pneumonia [27,28]. Furthermore, we provide a set of cutoffs for PCT for detection or exclusion of bacterial infection in ICU patients with potential 2009 H1N1 influenza. Three of the earlier studies did not report any cutoff, owing to weak overall discrimination of PCT or small sample size, and the two largest studies reported a cutoff of 0.8 μg/L. This cutoff is in between the generally accepted levels in ICU patients with respiratory tract infections [29], and the generalizability might be limited by the small and selected derivation sample. We show that recommended rule-out and rule-in cutoffs (0.25, 0.5 and 1 μg/L) for bacterial infection and, as a consequence, for antibiotic treatment discriminate moderately in patients with suspected 2009 H1N1 influenza infection. In patients with community-acquired disease without immune-compromising disorder, PCT might be helpful in ruling out bacterial infection or coinfection, with a negative predictive value of 82.2% and a negative likelihood ratio of 0.27. However, this negative predictive value still seems too low to use PCT as a stand-alone marker for withholding antibiotic therapy in such critically ill patients. Serial changes in PCT might further improve discrimination of bacterial pneumonia, but we had serial PCT measures in only 40% of our population, which is not sufficient for analysis. Thus, further study is needed to evaluate the clinical benefit of PCT testing and to define its role in an algorithm for antimicrobial therapy in ICU patients with suspected H1N1 influenza pneumonia.

In our patient population, PCT showed better discrimination than CRP for bacterial infection, albeit the difference was of borderline significance because of the small sample size. This finding is in line with a body of evidence supporting the superiority of PCT over CRP for detection of bacterial infection [30]. PCT is upregulated through stimulation by cytokines released in response to bacterial infection, and, in contrast to CRP, is inhibited through interferon γ, which is released in viral infections, thus exhibiting a strong specificity for bacterial infections [31].

Our study has some limitations. Despite the combination of six cohorts, our total sample size is still limited and thus subgroup analyses must be interpreted cautiously. Furthermore, half of the studies were retrospective and the study populations were variably selected, which limit the generalizability of our results and might have introduced bias. For instance, one-third of patients did not have PCT measured within the first 24 hours after admission. However, we show that these patients with missing PCT data were not significantly different from the patients included in the analysis regarding most baseline characteristics. A major limitation, which applies to almost all observational studies evaluating PCT, is that there is no accurate gold standard for the outcome definition of bacterial infection. The intensity of diagnostics have differed across studies and was clinically driven in all studies but ours and in the one by Piacentini *et al*. [20], who performed systematic microbiological testing on blood, urinary and tracheobronchial secretions in all patients. Nonetheless, microbiological cultures of bacterial pathogens are generally inaccurate, particularly in cases of respiratory infections [32,33]. Thus, underdiagnosing of bacterial infection might be common and thus might attenuate the resulting accuracy of PCT as a marker. Hence, to definitively assess the value of PCT in patients with possible H1N1 infection, a randomized trial on the clinical outcome of PCT-guided antimicrobial treatment is needed. Furthermore, our findings are not generalizable to other clinical scenarios, such as in the emergency department and cases of interpandemic pneumonia or other viral strains causing pneumonia, because the inflammatory response, including PCT, might differ, depending on clinical severity and the underlying viral pathogen [34]. We do not have information on the time from symptom onset to admission to the ICU, which might affect not only the risk of bacterial coinfection but also absolute PCT levels because the kinetics of PCT differ between viral and bacterial infections [20]. Hence the discriminatory performance of PCT might vary over time since the onset of infection. This factor might lead to underestimation of overall PCT accuracy.

## Conclusions

In patients admitted to the ICU with suspected H1N1 pneumonia, PCT is a sensitive marker with good negative predictive value for the identification of bacterial infection and is superior to CRP. The accuracy of PCT was highest in patients with community-acquired disease and in patients who are not immunocompromised. However, a negative predictive value of 82% might not be sufficient to use PCT as a stand-alone marker for withholding antibiotic treatment, and thus further studies are needed to define the clinical benefit of PCT testing in ICU patients during H1N1 epidemics.

## Key messages

• PCT shows good sensitivity and negative predictive value for bacterial pneumonia in critically ill patients during H1N1 influenza epidemics.

• The discrimination accuracy of PCT was best in patients with community-acquired pneumonia and in patients without immune-compromising disorders.

• The negative predictive value of PCT for bacterial pneumonia might not be sufficient to use it as a stand-alone marker for withholding antibiotics.

## Abbreviations

CRP: C-reactive protein; PCT: Procalcitonin; ROC: Receiver operating characteristic curve.

## Competing interests

The authors declare that they have no competing interests.

## Authors’ contributions

RP, MK and GM conceived of the study, participated in its design and coordination and drafted the manuscript. MK and TL designed the prospective cohort study in Cologne, screened patients and acquired and analyzed the data. RP, MK, TL and GM performed statistical analyses. CBB, EC, MBPM, EP, NH and PI acquired and analyzed data from the individual cohorts. TL, CBB, EC, MBPM, EP, NH and PI critically revised the manuscript for important intellectual content. All authors read and approved the final manuscript.

## Supplementary Material

Additional file 1 **Boxplot showing procalcitonin levels in isolated H1N1, isolated bacterial pneumonia and mixed bacterial and H1N1 pneumonia.** Procalcitonin levels are significantly increased in patients with isolated bacterial pneumonia and mixed bacterial and H1N1 pneumonia compared to patients with isolated H1N1 pneumonia, with no significant difference between patients with isolated bacterial pneumonia and mixed bacterial and H1N1 pneumonia. Click here for file
