# Supplementary figures and images for: Procalcitonin for diagnosis of bacterial pneumonia in critically ill patients during 2009 H1N1 influenza pandemic: a prospective cohort study, systematic review and individual patient data meta-analysis
Source: Crit Care. 2014 Mar 10;18(2):R44. doi: 10.1186/cc13760 (PMC4056761; doi:10.1186/cc13760)

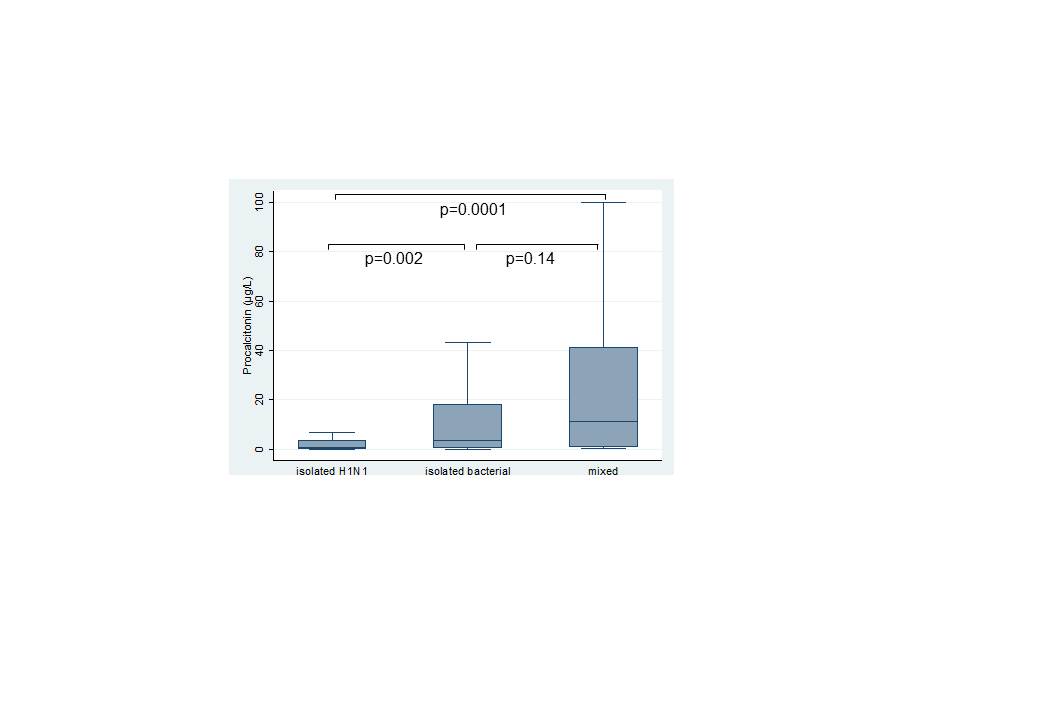

Supplement: Additional file 1 — Boxplot showing procalcitonin levels in isolated H1N1, isolated bacterial pneumonia and mixed bacterial and H1N1 pneumonia. Procalcitonin levels are significantly increased in patients with isolated bacterial pneumonia and mixed bacterial and H1N1 pneumonia compared to patients with isolated H1N1 pneumonia, with no significant difference between patients with isolated bacterial pneumonia and mixed bacterial and H1N1 pneumonia. [file cc13760-S1.jpeg]
